# Identifying the informational needs and sources of support of Adolescent and Young Adult (AYA) cancer survivors to inform the development of a digital platform

**DOI:** 10.1007/s11764-024-01679-z

**Published:** 2024-10-18

**Authors:** Carla Vlooswijk, Silvie H. M. Janssen, Sophia H. E. Sleeman, Jonas Pluis, Winette T. A. van der Graaf, Lonneke V. van de Poll-Franse, Olga Husson, Mies C. van Eenbergen

**Affiliations:** 1 Department of Research, Netherlands Comprehensive Cancer Organisation, Utrecht, the Netherlands; 2 Department of Psychosocial Research and Epidemiology, Netherlands Cancer Institute, Amsterdam, the Netherlands; 3 Department of Medical Oncology, Netherlands Cancer Institute – Antoni van Leeuwenhoek, Amsterdam, the Netherlands; 4 Dutch AYA ‘Young & Cancer’ Care Network, Utrecht, the Netherlands; 5 Department of Medical Oncology, Erasmus MC Cancer Institute, Erasmus University Medical Center, Rotterdam, the Netherlands; 6 Center of Research On Psychology in Somatic Diseases, Department of Medical and Clinical Psychology, Tilburg University, Tilburg, the Netherlands; 7 Department of Surgical Oncology, Erasmus MC Cancer Institute, Erasmus University Medical Center, Rotterdam, the Netherlands

**Keywords:** Adolescents and young adults (AYAs), Sources of support, Informational needs, Cancer

## Abstract

**Purpose:**

This study aimed to examine the (age-specific) informational needs and support sources used by Adolescent and Young Adult (AYA) cancer survivors throughout their cancer trajectory and socio-demographic and clinical factors associated with most common AYA-related informational needs.

**Methods:**

A cross-sectional questionnaire study was conducted among AYA cancer survivors (mean, 10.3 years after diagnosis, SD = 5.6). Informational needs and sources of support were examined via open questions and analyzed via a thematic inductive approach. Responses on informational needs were categorized according to the AYA anamnesis of the Dutch AYA “Young & Cancer” Care Network used in clinical practice. Chi-square and ANOVA tests were performed to assess differences in socio-demographic and clinical characteristics among AYA cancer survivors based on their varying levels of informational needs.

**Results:**

In total, 593 AYA cancer survivors were included (mean, 32.2 years at diagnosis, SD = 5.6). Most common informational needs were related to: family and children (23%), fertility and pregnancy (23%), work and reintegration (20%), peers with cancer (13%), and intimacy and sexuality (13%). Females, AYA cancer survivors diagnosed a longer time ago, those with a college/university education, those diagnosed with breast or hematological malignancies, and those treated with chemotherapy were more likely to have AYA-related informational needs. The most often used sources of support were healthcare professionals (76%), family (72%), social life (69%), and websites (47%).

**Conclusions:**

AYA cancer survivors have informational needs related to their life stage including topics like family and children, and fertility. Tailored information services and support are needed, including opportunities to connect with peers and support for relatives. By addressing the informational needs and sources of support for AYA cancer survivors, we can improve AYA care programs and empower AYA cancer survivors to better cope with the consequences associated with their disease.

**Implications for Cancer Survivors:**

This study will help to inform the content of AYA websites and platforms and help AYA cancer survivors, relatives, and healthcare professionals to become more aware of the needs of AYA cancer survivors and facilitate better use of relevant information and support services.

**Supplementary Information:**

The online version contains supplementary material available at 10.1007/s11764-024-01679-z.

## Introduction

A cancer diagnosis during Adolescence and Young Adulthood is particularly challenging due to the many emotional, cognitive, and social transitions that are taking place. In this phase of life, people are becoming independent from their parents, forming their own identity, becoming more active in both intimate and emotional relationships, finishing their education, starting careers, and having children [1, 2]. Treatment and its consequences can make Adolescents and Young Adults (AYAs) become more dependent on their parents (again); interrupt their studies, work, and/or social life; and potentially lead to loss of fertility [3].

The US Institute National Institute defined AYAs as individuals diagnosed with cancer between ages 15 and 39 years. This definition can be applied flexibly depending on the healthcare system. For instance, in the Netherlands, cancer care is divided between pediatric oncology for children (0–18 years) and medical oncology for adults (18 years and older). Consequently, the age range of 18–39 years is used as the definition of AYA cancer in this manuscript. AYA cancer survivors refer to individuals from the time of their cancer diagnosis and for the remainder of their life [4]. This phase can range from the initial diagnosis and treatment to post-treatment and long-term survivorship.

Current health services do not adequately meet the needs of many AYA cancer survivors, as highlighted by reviews on unmet needs, care experiences, and support services for AYA cancer survivors [5–8]. These unmet needs were related to the lack of age-appropriate facilities and healthcare professionals (HCPs) with expertise in AYA-specific issues. AYAs also identified gaps in care services, particularly concerning psychological support and social needs, including areas such as peer support. Unmet needs were found both during treatment [9–11] or after treatment [5, 8–10, 12, 13]. The unmet needs of recently diagnosed AYA cancer survivors were more often related to experiences with healthcare provision, whereas the unmet needs of AYA cancer survivors who had been treated for more than 1 year ago were centered around emotional and or psychological challenges [5].

Adequate individualized information provision is a commonly reported unmet need among AYA cancer survivors [5, 7–9, 14], with two thirds of them indicating medium to high levels of unmet informational needs [14]. Associations between unmet information needs and socio-demographic and clinical factors are poorly understood. Some studies indicate that women and younger age at diagnosis have more unmet information and healthcare needs [12, 13, 15, 16]. The informational needs of AYA cancer survivors seemed to partly overlap with young adult childhood cancer survivors. Recent research found that Dutch young adult childhood cancer survivors reported support needs for information related to lifestyle, fertility, and physical consequences of childhood cancer [17]. Compared to older cancer survivors, AYA cancer survivors have a greater need for information and needs differ in terms of content [18, 19]. AYA cancer survivors searched more often for information about fertility, lifestyle, sexuality and intimacy, insurance, treatment guidelines, end of life, and alternative or complementary therapies, whereas older adult cancer survivors searched more often for information about types of cancer, cancer genetics and heredity, financial problems, and opportunities to meet peers.

HCPs, family members and friends are all well-known sources of information and/or support [20]. Moreover, religious beliefs and hobbies can also be supportive in managing and coping with cancer. Nowadays, health information is widely accessible due to the internet and new technologies [21]. AYA cancer survivors belong to a generation that has grown up in a society shaped by the Internet. Consequently, cancer survivors are increasingly using digital sources for information and social support. Studies have recommended the improvement of reputable (Internet) resources specifically for young people in the AYA cancer care worldwide [8, 9, 22]. In the Netherlands, AYA cancer survivors, researchers, HCPs, and other relevant stakeholders have joined forces to improve online information and services for AYA cancer survivors throughout the whole cancer continuum, as part of a research infrastructure project funded by the Dutch Cancer Society [23]. This collaboration has led to the creation of an online “young and cancer” platform [24]. Here, cancer survivors can reflect on their experiences, compare their own patient-reported outcomes with those of other patients and peers, connect with each other, and receive age-specific support. Providing AYA cancer survivors with easy access to relevant and comprehensive information contributes to a better health-related quality of life and reducing anxiety and depression [19, 25]. To achieve this, the development of the platform involved gathering data on informational needs and sources of support used by AYA cancer survivors. Here, we present the results of this exploratory study into (1) the (age-specific) informational needs of AYA cancer survivors, (2) the socio-demographic and clinical factors associated with specific informational needs, and (3) the sources of support used by AYA cancer survivors during their cancer trajectory.

## Method

### Setting and study population

**Fig. 1:**
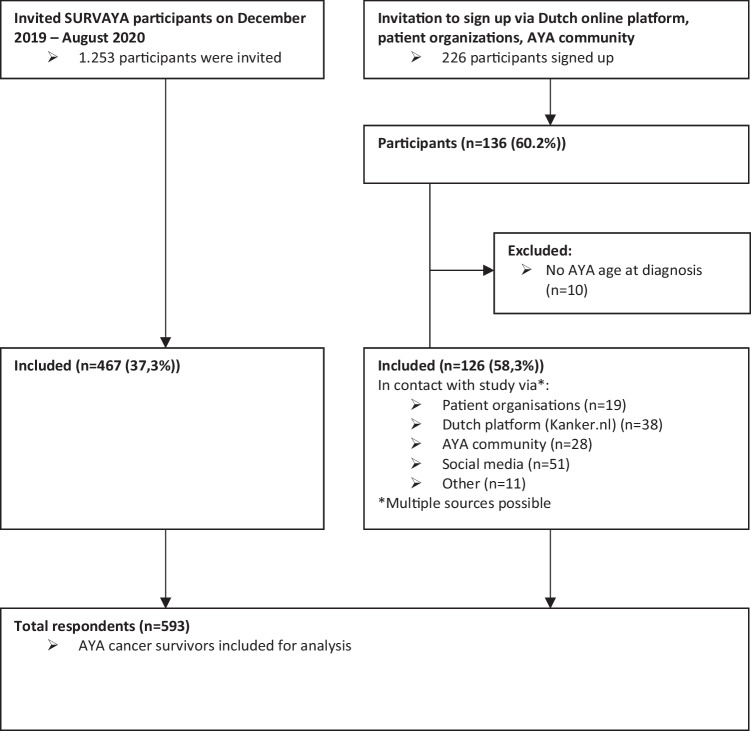
Flowchart of the data collection process

To improve online resources for AYA cancer survivors, an online “young and cancer” platform is being developed. We therefore conducted a preliminary research among AYA cancer survivors to investigate where they get their information from, and what information they needed during their illness. Therefore, a cross-sectional questionnaire study was conducted among AYA cancer survivors. In the Netherlands, care for cancer patients is divided into centralized pediatric oncology for children (0–18 years) and medical oncology for adults (≥ 18 years); therefore, AYA cancer survivors, aged 18 to 39 years old at cancer diagnosis were recruited [26]. They were recruited via the Dutch online platform for cancer survivors (Kanker.nl [27]), patient organizations, and the online AYA community (Fig. 1). They were directed to a webpage where they could sign up to receive information about the study and the informed consent procedure for participation. In addition, AYA cancer survivors who had already participated in the population-based SURVAYA study were invited to participate in this questionnaire study. The SURVAYA study focused on long-term (5–20 years) AYA cancer survivors and was conducted at the University Medical hospitals and the Netherlands Cancer Institute in the Netherlands [28]. Participants who responded to the SURVAYA study at time of this study and indicated that they could be contacted for follow-up research were invited to participate in this study. The current research was approved by the Research Ethics Review Committee of the Tilburg School of Humanities and Digital Sciences (internal code: REDC 2019.104). Cancer survivors were included who were able to read and write in Dutch and who did not have cognitive impairments that would hinder their ability to understand and complete the questionnaire, independently.

### Data collection

Data collection was conducted between December 2019 and August 2020 via the PROFILES (Patient Reported Outcomes Following Initial treatment and Long-term Evaluation of Survivorship) registry [29]. PROFILES is a registry to study the impact of a cancer diagnosis and treatment in a population-based cohort of survivors. AYA cancer survivors were able to register themselves for participation in the study via the PROFILES website. After registration, they received an e-mail with a secured link to the online informed consent and questionnaire. Participants of the SURVAYA study received a study invitation by email. In case no response was received after 3 weeks, a reminder email was sent. The information about the study and the questionnaire was sent out in the Dutch language.

### Measures

Socio-demographic characteristics (i.e., gender, age, marital status, children, and educational level) and clinical characteristics (year of diagnosis, tumor characteristics, and treatment modalities) were patient-reported via the questionnaire. Age-specific informational needs were assessed by the open-ended question: “What specific age-tailored information would you have wanted to receive, regarding your cancer diagnosis during adolescence and young adulthood?” Sources of support during AYA cancer survivors’ cancer trajectory were examined by the open-ended question: “What helped you the most during your illness? Consider people, websites, social media, apps, books, etc.” Respondents were asked to report anything that came to mind in open-ended text boxes, to a maximum of ten sources or topics, and to list the most important topics first. They were asked to reflect on these questions regarding their entire cancer continuum.

### Statistical analyses

The open-ended responses on age-specific informational needs and sources of support were categorized independently by two researchers (C.V. and S.J.) based on the Dutch AYA anamnesis designed by the Dutch AYA “Young & Cancer” Care Network, together with AYA cancer survivors and HCPs (Supplementary file 1). This AYA anamnesis tool is used in the preparation and/or conduction of age-specific consultations to facilitate the identification and monitoring of AYA cancer survivors’ care needs throughout the trajectory. The AYA anamnesis contains 18 topics: fertility and pregnancy, illness and treatments, education, work and reintegration, nutrition, appearance, sport and movement, emotions, spirituality, intimacy and sexuality, friends, family and children, benefits and compensations, mortgages and insurances, late effects, complementary care, palliative care, and death. An inductive approach was used, meaning new (sub)themes were created if needed, based on the data. In line with Janssen et al., the open-ended responses on informational needs were categorized using the themes of the AYA anamnesis [30]. Multiple topics/sources could be selected for each open-ended response. A third independent researcher (O.H.) was consulted to discuss discrepancies and reach agreement. When consensus was reached, C.V. finalized the categorization.

Statistical analyses were conducted using SAS version 9.4 (SAS Institute, Cary, NC, USA, 1999). Two-sided *P*-values of < 0.05 were considered statistically significant. The characteristics of the study population were presented as numbers and percentages or as means with standard deviations. Chi-square and ANOVA tests were conducted to examine the differences in socio-demographic and clinical characteristics between AYA cancer survivors with no informational needs, those with an informational need regarding one theme, and those with informational needs across multiple themes.

Chi-square and independent samples *t*-test were used to examine the socio-demographic and clinical characteristics of the most common AYA-related informational needs identified in our study and in the literature (fertility and pregnancy, family and children, work and reintegration, peers with cancer, intimacy and sexuality, mortgages and insurances, and appearance) [10, 11, 30, 31],

## Results

The data collection process for this questionnaire study is shown in Fig. 1. The majority of the participants already participated in the SURVAYA study (*n* = 467), while 126 participants were recruited via the Dutch online platform (Kanker.nl [27]), patient organizations and the online AYA community. Ten participants were excluded from the analyses as they did not meet the AYA age criteria at the time of their cancer diagnosis. Consequently, a total of 593 AYA cancer survivors participated in this questionnaire study.

The majority of the participants were female (70.8%) and the mean age at time of diagnosis was 32 years (Table 1). Almost half of the participants were more than 10 years of post-diagnosis (48.9%) and the majority had obtained college/university education (63.6%). The most common cancer diagnoses among the participants included breast cancer (32.7%), hematological malignancies (20.2%), and gynecological cancers (11.1%).

**Table 1:** Socio-demographic and clinical characteristics of the participants (*n* = 593)

		AYA cancer survivors (*n* = 593)					
			No informational needs	1 informational need	2 informational needs	> 2 informational needs	
		(*n* = 593)	(*n* = 21, 4.4%)	(*n* = 114, 23.7%)	(*n* = 115, 23.9%)	(*n* = 231, 48.0%)	
		*n* (%)	*n* (%)	*n* (%)	*n* (%)	*n* (%)	*P*-value
Sex	Female	420 (70.8%)	12 (57.1%)	73 (64.0%)	80 (69.6%)	190 (82.3%)	0
	Male	173 (29.2%)	9 (42.9%)	41 (36.0%)	35 (30.4%)	41 (17.7%)	
Age (at diagnosis), (mean (SD))		32.2 (5.6)	34.5 (3.8)	33.1 (5.3)	32.0 (5.7)	31.7 (5.6)	0.03862
	18–24 years	76 (12.8%)	1 (4.8%)	11 (9.6%)	15 (13.0%)	33 (14.3%)	0.29781
	25–32 years	184 (31.0%)	4 (19.0%)	32 (28.1%)	37 (32.2%)	78 (33.8%)	
	33–39 years	333 (56.2%)	16 (76.2%)	71 (62.3%)	63 (54.8%)	120 (51.9%)	
Years since diagnosis (mean (SD))		10.3 (5.6)	11.1 (3.6)	12.2 (4.9)	10.8 (5.4)	8.7 (5.6)	< .00012
	0–5 year(s)	105 (17.7%)	1 (4.8%)	10 (8.8%)	14 (12.2%)	67 (29.0%)	< .00011
	> 5–10 years	198 (33.4%)	9 (42.9%)	30 (26.3%)	41 (35.7%)	80 (34.6%)	
	> 10 years	290 (48.9%)	11 (52.4%)	74 (64.9%)	60 (52.2%)	84 (36.4%)	
Highest obtained educational level	Primary school or equivalent	1 (0.2%)	0 (0.0%)	0 (0.0%)	0 (0.0%)	0 (0.0%)	0.00071
	Secondary school or equivalent	214 (36.2%)	11 (52.4%)	54 (47.8%)	41 (35.7%)	63 (27.3%)	
	College/university or equivalent	376 (63.6%)	10 (47.6%)	59 (52.2%)	74 (64.3%)	168 (72.7%)	
Marital status (at time of questionnaire)	Partner	483 (81.5%)	18 (85.7%)	96 (84.2%)	96 (83.5%)	182 (78.8%)	0.52651
Having children		371 (62.6%)	15 (71.4%)	77 (67.5%)	78 (67.8%)	137 (59.3%)	0.25631
Type of cancer1	Brain cancer	32 (5.4%)	0 (0.0%)	8 (7.0%)	10 (8.7%)	9 (3.9%)	0.17031
	Breast cancer	194 (32.7%)	9 (42.9%)	34 (29.8%)	31 (27.0%)	91 (39.4%)	0.06861
	Bone and soft tissue sarcoma	23 (3.9%)	1 (4.8%)	7 (6.1%)	5 (4.3%)	4 (1.7%)	0.18791
	Gastrointestinal cancer	23 (3.9%)	0 (0.0%)	2 (1.8%)	3 (2.6%)	12 (5.2%)	0.26301
	Gynecological cancer	66 (11.1%)	0 (0.0%)	12 (10.5%)	16 (13.9%)	27 (11.7%)	0.31861
	Head and neck cancer	11 (1.9%)	1 (4.8%)	0 (0.0%)	3 (2.6%)	3 (1.3%)	0.22221
	Hematological malignancies	120 (20.2%)	3 (14.3%)	20 (17.5%)	30 (26.1%)	50 (21.6%)	0.18791
	Skin cancer	22 (3.7%)	1 (4.8%)	7 (6.1%)	3 (2.6%)	4 (1.7%)	0.15751
	Testicular cancer	56 (9.4%)	2 (9.5%)	13 (11.4%)	13 (11.3%)	15 (6.5%)	0.34121
	Thyroid cancer	27 (4.6%)	3 (14.3%)	8 (7.0%)	3 (2.6%)	8 (3.5%)	0.05111
	Urological cancer	10 (1.7%)	0 (0.0%)	2 (1.8%)	3 (2.6%)	4 (1.7%)	0.85561
	Other2	15 (2.5%)	1 (4.8%)	2 (1.8%)	1 (0.9%)	5 (2.2%)	0.63681
Treatment modality	Surgery	420 (70.8%)	14 (66.7%)	80 (70.2%)	78 (67.8%)	167 (72.3%)	0.82381
	Chemotherapy	397 (66.9%)	11 (52.4%)	71 (62.3%)	79 (68.7%)	175 (75.8%)	0.01821
	Radiotherapy	341 (57.5%)	13 (61.9%)	67 (58.8%)	56 (48.7%)	136 (58.9%)	0.27591
	Hormone therapy	116 (19.6%)	4 (19.0%)	26 (22.8%)	19 (16.5%)	53 (22.9%)	0.53971
	Immunotherapy	57 (9.6%)	1 (4.8%)	6 (5.3%)	10 (8.7%)	36 (15.6%)	0.01671
	Stem cell transplantation	35 (5.9%)	2 (9.5%)	5 (4.4%)	7 (6.1%)	14 (6.1%)	0.80311
	Targeted therapy	21 (3.5%)	3 (14.3%)	4 (3.5%)	2 (1.7%)	7 (3.0%)	0.03181
	Other3	13 (2.2%)	2 (9.5%)	2 (1.8%)	1 (0.9%)	5 (2.2%)	0.08541

^1^Participants can have multiple types of cancer

^2^Neuroendocrine tumor, lung cancer, mesothelioma, trophoblast tumor, multiple myeloma, esthesioneuroblastoma, thymus cancer and unknown

^3^Radio active iodine therapy and no therapy or active surveillance

### Informational needs

Data on (age-specific) informational needs were available from 481 AYA cancer survivors (Table 1). Among them, 21 AYA cancer survivors (4.4%) reported that they had no need for information. Nearly a quarter of the AYA cancer survivors, 23.7% reported informational needs related to a single theme, 23.9% reported informational needs on two themes, and nearly half of the AYA cancer survivors (48.0%) reported informational needs spanning more than two themes.

AYA cancer survivors with informational needs on multiple themes were more likely to be female (*p* < 0.001), younger at diagnosis (*p* = 0.04), more recently diagnosed with cancer (*p* < 0.001), obtained college/university education (*p* < 0.001), and more likely to be treated with chemotherapy (*p* = 0.02), immunotherapy (*p* = 0.02) and targeted therapy (*p* = 0.03) (Table 1).

Table 2 shows a detailed overview of the (sub)themes supported by corresponding quotes from AYA cancer survivors, which offer a deeper insight into the meaning of these (sub)themes. AYA cancer survivors most commonly reported a need for information within the *illness and treatment* theme (65.1%). AYA cancer survivors would have liked to have had information about treatment options, the pros and cons of various treatment, risks of recurrence, heredity, and survival rates. They also wanted information on how to find the best care, such as a list of hospitals specializing their specific type of cancer and age-appropriate care. Within this theme, two subthemes emerged. AYA cancer survivors also wanted information about what they can do themselves to support their recovery and rehabilitation options (aftercare and rehabilitation), as well as about the lasting side effects of the cancer diagnosis and treatment (short- and long-term effects).

*“How a young body reacts to chemotherapy and recovers from it, or what the residual symptoms are.”—*“Illness and treatment—short- and long-term effects” [female survivor of lymphoma]

**Table 2:** The (Sub)themes addressing the informational needs of AYA cancer survivors throughout their cancer trajectory

Themes	*n* (%)	Subtopics	*n* (%)	Quotes (EN)
Illness and treatment	313 (65.1%)			“Survival rates of young people”
				“What is the treatment procedure”
				“Overview of treatments with pros and cons”
				“Heredity”
				“Where can I find the best cancer care for this type of cancer”
				“Risk of recurrence, where to pay attention to”
				“How to be heard by a doctor, despite being young”
				“Treatment options tailored to young people”
		Aftercare and rehabilitation	54 (11.2%)	“Recovery period”
				“Which type of care is available”
				“How can I recover as best as possible”
		Short- and long-term effects	185 (38.5%)	“Long-term consequences”
				“Possible side effects of surgery and treatments”
				“Physical consequences of the disease and treatment”
				“Which complaints are associated with the consequences and which are not”
				“Energy after the treatment”
Overarching care and life	128 (26.6%)			“That life is no longer taken for granted”
				“Changes in your behaviour”
				“More positive stories, how things can go well with cancer”
		Information and guidance	27 (5.6%)	“Website were all information is available. For example where to buy a wig. Where to buy scarves? Where to buy customized bras. Everything in one place.”
				“Assistance with administration”
				“Step-by-step plan of what you need to arrange and what to consider, such as sperm bank/insurance”
				“Where to get support”
				“Which things are handy to arrange and in what timeline (wig, for example)”
				“Better information on what life is like as a cancer survivor”
		Life after cancer	79 (16.4%)	“What can you expect?”
				“Resuming daily life after treatment and acceptance”
Fertility and pregnancy	115 (23.9%)			“Risk of infertility”
				“Fertility in relation to treatments”
				“Desire to have children”
				“Options for (still) having children”
Family and children	112 (23.3%)			“How to best guide your children during the process. What can/should I tell them”
				“Cancer and its impact on young children within the family”
				“What to do when you are all alone with cancer within the family”
				“Disagreements within a family”
				“Support within the family”
				“How to deal with it in your relationship”
				“Household help (because I was young, I didn’t receive any assistance except for 3 h per week of childcare)”
Emotions	111 (23.1%)			“How do you deal with cancer, the uncertainty, the physical failure”
				“Recover from a positive self-image”
				“Emotional effects during curative treatment”
				“Anxiety after the illness (everything is suspicious)”
				“Mental consequences, psychological complaints”
				“How to deal with your ‘delay’ compared to others who continued with their lives”
				“Options for psychotherapy”
				“How to stay true to yourself in the web of advice, choiced, information, judgements, etc.”
Work and reintegration	98 (20.4%)			“Working and cancer”
				“What is the likelihood of fully returning to the workplace.”
				“Consequences for your job”
				“Returning to work when self-employed (there is a lot about returning to an employer, but not so much for self-employed individuals)”
				“Reintegration”
Friends	95 (19.8%)			“Friends”
				“How to deal with friends”
				“How to inform your friends and family”
				“How to involve your friends and family in such a process”
				“Interaction within friendships”
		Peers with cancer	61 (12.7%)	“How to find cancer patients of your own age close by”
				“How important physical contact with peer is”
				“Peers, do they exist?”
				“Contact with young peers”
				“Peer groups”
				“Experiences of peers with this disease”
		Social life	30 (6.2%)	“How do deal with being sick, feeling like you are not part of the society”
				“Return to society”
Intimacy and sexuality	58 (12.6%)			“More information on the impact on sexual possibilities after surgery”
				“Perhaps what effect it can have on intimacy in the relationship”
Mortgages and insurances	51 (10.6%)			“Influence of diagnosis on mortgage”
				“Options to buy a house with a non-removable cancer”
				“Mortgages and life insurance”
				“Insurance matters”
				“What are the consequences when applying for insurances/mortgage”
				“How can you get a mortgage/what alternatives are there in terms of housing”
				“Life insurance”
				“Finances (benefits, taxes (health care costs), mortgage, etc.)”
				“Financial consequences ((partners’) pension, life insurance, etc.)”
Late effects	39 (8.1%)			“Late effects”
				“Information regarding possible late effects in a age-appropriate manner”
				“Side effects at an older age”
Sport and movement	37 (7.7%)			“Long-term impact on your body during sports (fatigue/muscle problems/etc.)”
				“Reintroducing sports, my treating doctor and physiotherapist played a from pillar to post-game”
				“Good condition”
				“Recovery with exercise”
Nutrition	36 (7.5%)			“Nutritional advice”
				“Nutrition as preventive medication (vegan/dairy-free)”
Benefits and compensations	35 (7.3%)			“Income and cancer”
				“That it has financial consequences”
Appearance	22 (4.6%)			“How to hide physical features such as baldness”
				“Scar reconstruction”
				“Acceptance of body disfigurement”
				“Info on changes in appearance (hair loss, where to buy good wigs or scarves, make-up)”
Complementary care	10 (2.1%)			“Alternative medicine”
Spirituality	5 (1.0%)			“Religion and purpose”
				“Spirituality”
				“Finding meaning after cancer at a young age”
				“Spirituality after cancer”
Education	4 (0.8%)			“Studying with brain injury”
				“Assistance with returning to work/study”
				“Study and work after treatment”
				“How to deal with resuming to studies”
Death	2 (0.4%)			“Am I going to die?”
				“How can you prepare for your own death, and how do you involve your family in this?”
Palliative care	1 (0.2%)			“When are you considered as ‘exhausted all treatment options,’ and what can you still do after such a diagnosis”

Because many AYA cancer survivors reported informational needs that could not be categorized under one theme (i.e., not limited to a specific theme, but covering broader aspects), the *overarching care and life* theme (26.6%) was created. Many AYA cancer survivors expressed the need for information on how to get back on track after cancer and learn to cope with the consequences of cancer for the rest of their lives.

*“How to pick up the pieces after cancer, in a world where cancer seems to affect mainly older people.”—*“Overarching care and life”—[female cervix carcinoma survivor]

Within the overarching care and life theme, two subthemes emerged: one focused on the need for information and guidance, and the other addressed life after cancer.

*“A lot of information was tailored to an older target group.”—*“Overarching care and life—information and guidance” [female breast cancer survivor]

*‘What changes for me?”—*“Overarching care and life—life after cancer” [female breast cancer survivor]

Information on *fertility and pregnancy* (23.9%) focused on fertility in general or in relation to treatment, the potential risks on infertility after treatment, risks during pregnancy, and possibilities of conceiving or expanding their families. AYA cancer survivors also needed information related to *family and children* (23.3%). Information was needed on advice on communicating the cancer diagnosis and treatment to their children and impact on their relationship with their partner. In addition, practical support to keep the household running during treatment was also warranted. AYA cancer survivors reported that they missed (age-specific) information about psychological support during and after their diagnosis, as categorized into the *emotions* (23.1%) theme. They missed information about guidance in addressing their psychological complaints, including feelings of anxiety, fear of recurrence, and lack of positive self-image.

*“How to deal with frustrations when the things you did before are now no longer possible, and what you can do about it.”—*“Emotions” [female colorectal cancer survivor]

AYA cancer survivors have indicated the need for guidance and support regarding *work and reintegration* (20.4%). There was a lack of information about how their employment might be affected, how to communicate with colleagues, expectations regarding a full return to work, and information for AYA cancer survivors who are self-employed. AYA cancer survivors wanted to have information on how to inform and cope with their *friends* (19.8%) during their disease. AYA cancer survivors had the desire to share experiences with peers with cancer. On one hand, AYA cancer survivors desire guidance on how to reintegrate into social activities (again), while on the other hand, they have trouble to maintain connections with “healthy” peers.

*“Offer opportunity for contact with young people with cancer. (In the rehabilitation group, I was the only young person with cancer, all others were elderly.”—*“Friends—peers with cancer” [female thyroid cancer survivor]

AYA cancer survivors wanted to have information about *intimacy and sexuality* (12.6%). This included in particular, information on how their treatment and diagnosis affected intimacy and sexuality, tailored to the type of cancer and treatment received. Within the *mortgages and insurances* (10.6%) theme, AYA cancer survivors were concerned about the financial consequences of cancer. In particular, its effects on obtaining mortgages, (life) insurances, and pensions. They were worried about how cancer and its treatment might influence their ability to secure these financial necessities. Other themes that were mentioned by less than 10% of the AYA cancer survivors were *late effects, sport and movement, nutrition, benefits and compensations, appearance, complementary care, spirituality, education, death,* and *palliative care.*

### Socio-demographic and clinical characteristics associated with AYA *cancer* survivors’ informational needs

Table 3 shows the socio-demographic and clinical characteristics associated with AYA-related informational needs. Compared to men, female AYA cancer survivors were significantly more likely to have informational needs related to fertility and pregnancy, family and children, work and reintegration, intimacy, and sexuality and appearance. Younger AYA cancer survivors were significantly more likely to have informational needs related to fertility and pregnancy, peers with cancer, mortgages and insurances, and appearance, while older AYA cancer survivors were significantly more likely to have informational needs related to family and children. AYA cancer survivors who had more recently been diagnosed with cancer were significantly more likely to have informational needs regarding family and children, work and reintegration, peers with cancer, and intimacy and sexuality. AYA cancer survivors who obtained college/university education were significantly more likely to have informational needs regarding fertility and pregnancy, peers with cancer, intimacy and sexuality, and mortgages and insurances. AYA cancer survivors who had been treated with chemotherapy were significantly more likely to have informational needs on fertility and pregnancy, family and children, work and reintegration, and intimacy and sexuality.

**Table 3:** Socio-demographic and clinical characteristics of AYA cancer survivors with (age-specific) informational needs

		Fertility and pregnancy			Family and children			Work and reintegration			Peers with cancer		
		No informational needs	Informational needs		No informational needs	Informational needs		No informational needs	Informational needs		No informational needs	Informational needs	
		(*n* = 366)	(*n* = 115)		(*n* = 369)	(*n* = 1 12)		(*n* = 383)	(*n* = 98)		(*n* = 420)	(*n* = 61)	
		*N* (%)	*N* (%)		*N* (%)	*N* (%)		*N* (%)	*N* (%)	0.01411	*N* (%)	*N* (%)	
Sex	Female	**257 (70.2%)**	**98 (85.2%)**	*	**261 (70.7%)**	**94 (83.9%)**	*	273 (71.3%)	82 (83.7%)		306 (72.9%)	49 (80.3%)	
	Male	**109 (29.8%)**	**17 (14.8%)**		**108 (29.3%)**	**18 (16.1%)**		110 (28.7%)	16 (16.3%)		114 (27.1%)	12 (19.7%)	
Age at diagnosis (mean (SD))		**32.9 (5.5)**	**30.1 (5.1)**	*	**31.9 (5.9)**	**33.4 (4.1)**	*	32.2 (5.7)	32.6 (5.1)		**32.5 (5.4)**	**30.7 (6.3)**	*
	18–24 years	**42 (11.5%)**	**18 (15.7%)**	*	**58 (15.7%)**	**2 (1.8%)**	*	51 (13.3%)	9 (9.2%)		**46 (11.0%)**	**14 (23.0%)**	*
	25–32 years	**89 (24.3%)**	**62 (53.9%)**		**109 (29.5%)**	**42 (37.5%)**		119 (31.1%)	32 (32.7%)		**135 (32.1%)**	**16 (26.2%)**	
	33–39 years	**235 (64.2%)**	**35 (30.4%)**		**202 (54.7%)**	**68 (60.7%)**		213 (55.6%)	57 (58.2%)		**239 (56.9%)**	**31 (50.8%)**	
Years since diagnosis (mean (SD))		10.3 (5.44)	9.6 (5.66)		10.4 (5.29)	9.3 (6.07)		**10.5 (5.5)**	**8.8 (5.5)**	*	**10.5 (5.5)**	**7.7 (5.1)**	*
	0–5 year(s)	63 (17.2%)	29 (25.2%)		**59 (16.0%)**	**33 (29.5%)**	*	**65 (17.0%)**	**27 (27.6%)**	*	**74 (17.6%)**	**18 (29.5%)**	*
	> 5–10 years	127 (34.7%)	33 (28.7%)		**131 (35.5%)**	**29 (25.9%)**		**126 (32.9%)**	**34 (34.7%)**		**131 (31.2%)**	**29 (47.5%)**	
	> 10 years	176 (48.1%)	53 (46.1%)		**179 (48.5%)**	**50 (44.6%)**		**192 (50.1%)**	**37 (37.8%)**		**215 (51.2%)**	**14 (23.0%)**	
Highest obtained educational level	Primary school or equivalent	**0 (0%)**	**0 (0%)**	*	0 (0%)	0 (0%)		0 (0%)	0 (0%)		**0 (0%)**	**0 (0%)**	*
	Secondary school or equivalent	**146 (40.0%)**	**23 (20.0%)**		137 (37.2%)	32 (28.6%)		141 (36.9%)	28 (28.6%)		**155 (37.0%)**	**14 (23.0%)**	
	College/university or equivalent	**219 (60.0%)**	**92 (80.0%)**		231 (62.8%)	80 (71.4%)		241 (63.1%)	70 (71.4%)		**264 (63.0%)**	**47 (77.0%)**	
Marital status (at time of questionnaire)	Partner	295 (80.6%)	97 (84.3%)		**293 (79.4%)**	**99 (88.4%)**	*	314 (82.0%)	78 (79.6%)		342 (81.4%)	50 (82.0%)	
Having children		233 (63.7%)	74 (64.3%)		**216 (58.5%)**	**91 (81.3%)**	*	250 (65.3%)	57 (58.2%)		**279 (66.4%)**	**28 (45.9%)**	*
Type of cancer1	Brain cancer	24 (6.6%)	3 (2.6%)		20 (5.4%)	7 (6.3%)		20 (5.2%)	7 (7.1%)		21 (5.0%)	6 (9.8%)	
	Breast cancer	116 (31.7%)	49 (42.6%)		**115 (31.2%)**	**50 (44.6%)**	*	123 (32.1%)	42 (42.9%)		147 (35.0%)	18 (29.5%)	
	Bone and soft tissue sarcoma	16 (4.4%)	1 (0.9%)		12 (3.3%)	5 (4.5%)		16 (4.2%)	1 (1.0%)		17 (4.0%)	0 (0.0%)	
	Gastrointestinal cancer	16 (4.4%)	1 (0.9%)		9 (2.4%)	8 (7.1%)		12 (3.1%)	5 (5.1%)		13 (3.1%)	4 (6.6%)	
	Gynecological cancer	39 (10.7%)	16 (13.9%)		43 (11.7%)	12 (10.7%)		47 (12.3%)	8 (8.2%)		48 (11.4%)	7 (11.5%)	
	Head and neck cancer	7 (1.9%)	0 (0.0%)		7 (1.9%)	0 (0.0%)		**3 (0.8%)**	**4 (4.1%)**	*	5 (1.2%)	2 (3.3%)	
	Hematological malignancies	**69 (18.9%)**	**34 (29.6%)**	*	**87 (23.6%)**	**16 (14.3%)**	*	82 (21.4%)	21 (21.4%)		85 (20.2%)	18 (29.5%)	
	Skin cancer	14 (3.8%)	1 (0.9%)		14 (3.8%)	1 (0.9%)		14 (3.7%)	1 (1.0%)		14 (3.3%)	1 (1.6%)	
	Testicular cancer	32 (8.7%)	11 (9.6%)		38 (10.3%)	5 (4.5%)		**40 (10.4%)**	**3 (3.1%)**	*	39 (9.3%)	4 (6.6%)	
	Thyroid cancer	20 (5.5%)	2 (1.7%)		20 (5.4%)	2 (1.8%)		18 (4.7%)	4 (4.1%)		21 (5.0%)	1 (1.6%)	
	Urological cancer	8 (2.2%)	1 (0.9%)		7 (1.9%)	2 (1.8%)		8 (2.1%)	1 (1.0%)		9 (2.1%)	0 (0.0%)	
	Other2	9 (2.5%)	0 (0.0%)		5 (1.4%)	4 (3.6%)		8 (2.1%)	1 (1.0%)		8 (1.9%)	1 (1.6%)	
Treatment modality	Surgery	260 (71.0%)	79 (68.7%)		**251 (68.0%)**	**88 (78.6%)**	*	267 (69.7%)	72 (73.5%)		296 (70.5%)	43 (70.5%)	
	Chemotherapy	**247 (67.5%)**	**89 (77.4%)**	*	**249 (67.5%)**	**87 (77.7%)**	*	**258 (67.4%)**	**78 (79.6%)**	*	291 (69.3%)	45 (73.8%)	
	Radiotherapy	207 (56.6%)	65 (56.5%)		204 (55.3%)	68 (60.7%)		208 (54.3%)	64 (65.3%)		239 (56.9%)	33 (54.1%)	
	Hormone therapy	74 (20.2%)	28 (24.3%)		74 (20.1%)	28 (25.0%)		81 (21.1%)	21 (21.4%)		92 (21.9%)	10 (16.4%)	
	Immunotherapy	35 (9.6%)	18 (15.7%)		36 (9.8%)	17 (15.2%)		**36 (9.4%)**	**17 (17.3%)**	*	45 (10.7%)	8 (13.1%)	
	Stem cell transplantation	18 (4.9%)	10 (8.7%)		21 (5.7%)	7 (6.3%)		18 (4.7%)	10 (10.2%)		25 (6.0%)	3 (4.9%)	
	Targeted therapy	12 (3.3%)	4 (3.5%)		14 (3.8%)	2 (1.8%)		14 (3.7%)	2 (2.0%)		14 (3.3%)	2 (3.3%)	
	Other3	8 (2.2%)	2 (1.7%)		9 (2.4%)	1 (0.9%)		6 (1.6%)	4 (4.1%)		10 (2.4%)	0 (0.0%)	
Unmet information need	Fertility and pregnancy	**0 (0.0%)**	**115 (100.0%)**	*	83 (22.5%)	32 (28.6%)		91 (23.8%)	24 (24.5%)		101 (24.0%)	14 (23.0%)	
	Family and children	80 (21.9%)	32 (27.8%)		**0 (0.0%)**	**112 (100.0%)**	*	**75 (19.6%)**	**37 (37.8%)**	*	102 (24.3%)	10 (16.4%)	
	Work and reintegration	74 (20.2%)	24 (20.9%)		**61 (16.5%)**	**37 (33.0%)**	*	**0 (0.0%)**	**98 (100.0%)**	*	83 (19.8%)	15 (24.6%)	
	Peers with cancer	47 (12.8%)	14 (12.2%)		51 (13.8%)	10 (8.9%)		46 (12.0%)	15 (15.3%)		**0 (0.0%)**	**61 (100.0%)**	*
	Intimacy and sexuality	**38 (10.4%)**	**20 (17.4%)**	*	**35 (9.5%)**	**23 (20.5%)**	*	41 (10.7%)	17 (17.3%)		48 (11.4%)	10 (16.4%)	
	Mortgages and insurances	**31 (8.5%)**	**20 (17.4%)**	*	39 (10.6%)	12 (10.7%)		**29 (7.6%)**	**22 (22.4%)**	*	44 (10.5%)	7 (11.5%)	
	Appearance	**11 (3.0%)**	**11 (9.6%)**	*	15 (4.1%)	7 (6.3%)		15 (3.9%)	7 (7.1%)		18 (4.3%)	4 (6.6%)	

		Intimacy and sexuality			Mortgages and insurances			Appearance					
		No informational needs	Informational needs		No informational needs	Informational needs		No informational needs	Informational needs				
		(*n* = 423)	(*n* = 58)		(*n* = 430)	(*n* = 51)		(*n* = 459)	(*n* = 22)				
		*N* (%)	*N* (%)		*N* (%)	*N* (%)		*N* (%)	*N* (%)				
Sex	Female	**305 (72.1%)**	**50 (86.2%)**	*	317 (73.7%)	38 (74.5%)		**334 (72.8%)**	**21 (95.5%)**	*			
	Male	**118 (27.9%)**	**8 (13.8%)**		113 (26.3%)	13 (25.5%)		**125 (27.2%)**	**1 (4.5%)**				
Age at diagnosis (mean (SD))		32.2 (5.4)	32.4 (6.5)		**32.5 (5.4)**	**30.2 (6.4)**	*	**32.4 (5.5)**	**29.5 (6.2)**	*			
	18–24 years	52 (12.3%)	8 (13.8%)		**48 (11.2%)**	**12 (23.5%)**	*	**55 (12.0%)**	**5 (22.7%)**	*			
	25–32 years	136 (32.2%)	15 (25.9%)		**132 (30.7%)**	**19 (37.3%)**		**141 (30.7%)**	**10 (45.5%)**				
	33–39 years	235 (55.6%)	35 (60.3%)		**250 (58.1%)**	**20 (39.2%)**		**263 (57.3%)**	**7 (31.8%)**				
Years since diagnosis (mean (SD))		**10.4 (5.4)**		*	10.2 (5.5)	10.0 (5.7)		10.2 (5.5)	8.0 (5.1)				
	0–5 year(s)	**72 (17.0%)**	**20 (34.5%)**	*	81 (18.8%)	11 (21.6%)		85 (18.5%)	7 (31.8%)				
	> 5–10 years	**140 (33.1%)**	**20 (34.5%)**		143 (33.3%)	17 (33.3%)		153 (33.3%)	7 (31.8%)				
	> 10 years	**211 (49.9%)**	**18 (31.0%)**		206 (47.9%)	23 (45.1%)		221 (48.1%)	8 (36.4%)				
Highest obtained educational level	Primary school or equivalent	**0 (0%)**	**0 (0%)**	*	**0 (0%)**	**0 (0%)**	*	0 (0%)	0 (0%)				
	Secondary school or equivalent	**157 (37.2%)**	**12 (20.7%)**		**160 (37.3%)**	**9 (17.6%)**		163 (35.6%)	6 (27.3%)				
	College/university or equivalent	**265 (62.8%)**	**46 (79.3%)**		**269 (62.7%)**	**42 (82.4%)**		295 (64.4%)	16 (72.7%)				
Marital status (at time of questionnaire)	Partner	349 (82.5%)	43 (74.1%)		350 (81.4%)	42 (82.4%)		377 (82.1%)	15 (68.2%)				
Having children		275 (65.0%)	32 (55.2%)		275 (64.0%)	32 (62.7%)		296 (64.5%)	11 (50.0%)				
Type of cancer1	Brain cancer	25 (5.9%)	2 (3.4%)		24 (5.6%)	3 (5.9%)		26 (5.7%)	1 (4.5%)				
	Breast cancer	139 (32.9%)	26 (44.8%)		153 (35.6%)	12 (23.5%)		**152 (33.1%)**	**13 (59.1%)**	*			
	Bone and soft tissue sarcoma	15 (3.5%)	2 (3.4%)		15 (3.5%)	2 (3.9%)		17 (3.7%)	0 (0.0%)				
	Gastrointestinal cancer	14 (3.3%)	3 (5.2%)		15 (3.5%)	2 (3.9%)		16 (3.5%)	1 (4.5%)				
	Gynecological cancer	**42 (9.9%)**	**13 (22.4%)**	*	51 (11.9%)	4 (7.8%)		55 (12.0%)	0 (0.0%)				
	Head and neck cancer	7 (1.7%)	0 (0.0%)		7 (1.6%)	0 (0.0%)		7 (1.5%)	0 (0.0%)				
	Hematological malignancies	96 (22.7%)	7 (12.1%)		**86 (20.0%)**	**17 (33.3%)**	*	97 (21.1%)	6 (27.3%)				
	Skin cancer	15 (3.5%)	0 (0.0%)		13 (3.0%)	2 (3.9%)		14 (3.1%)	1 (4.5%)				
	Testicular cancer	40 (9.5%)	3 (5.2%)		40 (9.3%)	3 (5.9%)		43 (9.4%)	0 (0.0%)				
	Thyroid cancer	22 (5.2%)	0 (0.0%)		20 (4.7%)	2 (3.9%)		22 (4.8%)	0 (0.0%)				
	Urological cancer	7 (1.7%)	2 (3.4%)		9 (2.1%)	0 (0.0%)		9 (2.0%)	0 (0.0%)				
	Other2	9 (2.1%)	0 (0.0%)		7 (1.6%)	2 (3.9%)		9 (2.0%)	0 (0.0%)				
Treatment modality	Surgery	293 (69.3%)	46 (79.3%)		307 (71.4%)	32 (62.7%)		325 (70.8%)	14 (63.6%)				
	Chemotherapy	**287 (67.8%)**	**49 (84.5%)**	*	300 (69.8%)	36 (70.6%)		317 (69.1%)	19 (86.4%)				
	Radiotherapy	237 (56.0%)	35 (60.3%)		241 (56.0%)	31 (60.8%)		258 (56.2%)	14 (63.6%)				
	Hormone therapy	**81 (19.1%)**	**21 (36.2%)**	*	95 (22.1%)	7 (13.7%)		**93 (20.3%)**	**9 (40.9%)**	*			
	Immunotherapy	**42 (9.9%)**	**11 (19.0%)**	*	46 (10.7%)	7 (13.7%)		**47 (10.2%)**	**6 (27.3%)**	*			
	Stem cell transplantation	25 (5.9%)	3 (5.2%)		22 (5.1%)	6 (11.8%)		26 (5.7%)	2 (9.1%)				
	Targeted therapy	15 (3.5%)	1 (1.7%)		15 (3.5%)	1 (2.0%)		16 (3.5%)	0 (0.0%)				
	Other3	10 (2.4%)	0 (0.0%)		8 (1.9%)	2 (3.9%)		10 (2.2%)	0 (0.0%)				
Unmet information need	Fertility and pregnancy	**95 (22.5%)**	**20 (34.5%)**	*	**95 (22.1%)**	**20 (39.2%)**	*	**104 (22.7%)**	**11 (50.0%)**	*			
	Family and children	**89 (21.0%)**	**23 (39.7%)**	*	100 (23.3%)	12 (23.5%)		105 (22.9%)	7 (31.8%)				
	Work and reintegration	81 (19.1%)	17 (29.3%)		**76 (17.7%)**	**22 (43.1%)**	*	91 (19.8%)	7 (31.8%)				
	Peers with cancer	51 (12.1%)	10 (17.2%)		54 (12.6%)	7 (13.7%)		57 (12.4%)	4 (18.2%)				
	Intimacy and sexuality	**0 (0.0%)**	**58 (100.0%)**	*	50 (11.6%)	8 (15.7%)		54 (11.8%)	4 (18.2%)				
	Mortgages and insurances	43 (10.2%)	8 (13.8%)		**0 (0.0%)**	**51 (100.0%)**	*	48 (10.5%)	3 (13.6%)				
	Appearance	18 (4.3%)	4 (6.9%)		19 (4.4%)	3 (5.9%)		0 (0.0%)	22 (100.0%)				

^1^Participants can have multiple types of cancer

^2^Neuroendocrine tumor, mesothelioma, trophoblast tumor, multiple myeloma, esthesioneuroblastoma, thymus cancer, and unknown

^3^Radio active iodine therapy and no therapy or active surveillance

^*^p < 0.05

### Sources of support

**Fig. 2:**
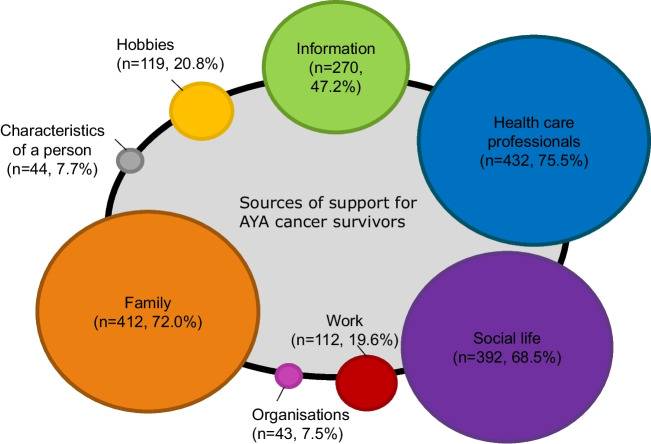
Sources of support utilized by AYA cancer survivors during their cancer continuum

In total, 472 AYA cancer survivors listed up to ten sources of support in open-ended textboxes, which are graphically displayed in Fig. 2. HCPs (75.5%), family (72.0%), and social life (68.5%) were most often reported as important sources of support during the illness of the AYA, followed by information (47.2%), hobbies (20.8%), work (19.6%), characteristics of a person (7.7%), and organizations (7.5%). Within the HCPs category, physicians were cited most often (58.7%), followed by nurses (29.9%), and other care givers (12.6%), such as psychotherapists, social workers, dietitians, occupational physicians, and pharmacists. In the family category, (ex-)partners were most often mentioned (32.0%), followed by parents (in law) (15.2%), and children (10.5%). Friends (46%) and peers with cancer (22.9%) were the most frequently mentioned sources of support in the social life category. Almost half of the AYA cancer survivors reported information sources such as websites (35.0%) and medical literature (18.5%). Other sources of support were hobbies (20.8%) which included sport, relaxation exercises, mediation, mindfulness, yoga; spare time and recovery (9.1%), as well as social media, gaming, and watching television (7.9%). Within the work category, colleagues and the management team (14,9%) were the most frequently mentioned sources of support. Characteristics of a person, such as positive attitude and perseverance, were also considered as helpful.

## Discussion

In addition to common informational needs related to cancer and its treatment and psychological support, AYA cancer survivors reported a need for age-specific information related to fertility, family and children, work and reintegration, peers with cancer, and intimacy and sexuality. The need for information on AYA-related topics was greater among females, AYA cancer survivors diagnosed a longer time ago, those with a college/university education, those diagnosed with breast or hematological malignancies, and AYA cancer survivors who underwent chemotherapy. HCPs, family, social life, and information, including website and medical literature, were emerged as the most often used sources of information and support throughout their disease trajectory.

Previous research found that the most common informational needs among AYA cancer survivors were related to their disease and treatment aspects and the consequences of treatment, including late effects [10, 11, 14, 32–34]. These needs were also identified among older cancer survivors as well [35]. In our study, AYA cancer survivors reported that a lot of information is tailored to older cancer survivors and that they wanted information on age-specific aspects, such as information on treatment options specifically for young cancer survivors, risk on infertility, guidance on how to effectively support their children during their cancer trajectory, along with practical assistance in household tasks, and childcare. These findings support the need for psychological and appropriate services on these age-specific topics for AYA cancer survivors, especially after treatment is completed [5, 8, 36, 37]. AYA cancer survivors reported that certain characteristics, such as having a positive mindset, perseverance, the ability to put things into perspective, and knowledge about their disease were reported as helpful during their disease. Interventions that focus on enhancing these personal characteristics may potentially enable AYA cancer survivors to better cope with their disease.

Several characteristics were associated with the need for information on AYA-related topics. In our study, younger AYA cancer survivors were significantly more likely to report informational needs on multiple themes compared to older AYA cancer survivors. This was supported by Zebrack et al. who found that unmet needs were more likely to be reported by AYA cancer survivors who were younger at diagnosis [37]. Contradictory findings were observed in another study, where younger cancer survivors (15–19 years at cancer diagnosis) had fewer unmet informational needs than those aged 30–39 years [34]. This difference may be related to their predominantly male AYA study population, who also reported less informational needs in our study. Our study reported also that younger AYA cancer survivors were more likely to have informational needs about AYA-specific themes such as fertility and pregnancy, peers with cancer, mortgages and insurances, and appearance. While older AYA cancer survivors were more likely to have informational needs related to family and children. AYA cancer survivors who completed treatment have greater informational needs than those currently undergoing treatment [34]. The main concern of AYA cancer survivors who have completed treatment was that they wanted timely and comprehensive information about the lasting impact of cancer and its treatment. In our study, short-term AYA cancer survivors had significantly more often informational needs regarding family and children, work and reintegration, peers with cancer, and intimacy and sexuality, compared to long-term cancer survivors (> 5 years). Informational needs seem to be influenced by the stage of life at which AYA cancer survivors are when diagnosed, and this should be taken into consideration when addressing informational needs by offering different things on the platform.

In our study, HCPs, mainly the physician, nurse, and the treatment team, were most frequently identified as a support source for AYA cancer survivors. This is in line with previous studies on social support among AYA cancer survivors, in which hospital staff were considered an important source of social support, largely in terms of providing informational support [20]. The study of Bennett and colleagues showed that younger survivors (aged < 60 years at diagnosis) were more likely to seek information from various sources beyond their HCPs compared to older survivors [38]. Cancer survivors with a college/university degree have greater informational needs compared to those with no education or only a primary/secondary school background. Younger cancer patients tend to have more often a college or university education compared to older cancer patients, suggesting that they may need more information to meet their informational needs. This may have implication for information services in the future. AYA cancer survivors may feel more empowered and involved in their care by actively seeking information online and discussing their options with friends and family, which is in line with societal trends towards increased patient engagement and involvement. Advances in patient- and family-centered care that have an impact on how patients actively seek information and support from HCPs are, for example, digital patient portals. They provide access to medical records, test results, and treatment plans, as well as patient-reported outcomes tools, such as those tracking symptoms. Such tools provide valuable insights into the patient’s condition for both the cancer patient and the HCP, and help them to make informed decision about their treatment.

AYA cancer survivors who are supported by close relationships with family, friends, work, or other support groups experienced less distress. McNeil et al. found that parents provide emotional, informational and instrumental social support, even for older AYA cancer survivors and those with partners and children [20]. While older adult cancer survivors are often supported by their children, AYA cancer survivors typically rely on a different support system, such as parents or peers. This responsibility can weigh heavily on partners, family members, and peers of AYA cancer survivors and can potentially lead to physical and psychological problems [39, 40]. Relatives have their own needs, which go beyond just providing care for AYA cancer survivors. Therefore, it is important that interventions which address the needs of AYA cancer survivors include social support, but also recognize that the support system of the AYA may need support and understanding as they navigate the complexities of supporting an AYA with unique challenges.

In our study, only 8.1% of the AYA cancer survivors’ informational needs is related to late effects. The need for information on short- and long-term effects was reported more frequently (38.5%). In previous research, late effects were mentioned by almost 80% of the AYA cancer survivors [8, 32, 33]. An explanation for the difference in the percentage of AYA cancer survivors reporting informational needs regarding late effects could be that AYA cancer survivors may be unfamiliar with late effects and use the terms late effects and long-term effects interchangeably. The difference between them is that late effects appear months or years after the end of the treatment or diagnosis, whereas long-term effects persist for a longer period after treatment. Moreover, it could be attributed to the used definition of late effects (e.g., fertility included or not). Information on late effects is highly prioritized by childhood cancer survivors. They indicated that they would visit a website with information on late effects with personalized information, follow-up strategies, and preventive healthcare information. Further research should be conducted to gain more insight in how long-term follow-up care for AYA cancer survivors can be organized. Survivorship care plans are currently used to inform, support communication, and facilitate self-care of AYA cancer survivors regarding cancer diagnosis and follow-up [33]. Apart from providing information on medical treatment and follow-up care, these plans could be personalized, based on individual characteristics, and include details on the risk of long-term and late effects, as well as address the emotional and psychological impact. They should include a point of contact for age-specific issues, serving as navigators, referring AYA cancer survivors to appropriate HCPs. More research is needed on how these plans could be personalized based on individual characteristics using digital technologies and their impact on health outcomes.

### Strength and limitations

The strength of this study is the various recruitment methods used to obtain a diverse AYA sample and thereby including a large number of AYA cancer survivors, particularly those who are long-term survivors (> 5 years of post-cancer diagnosis). Furthermore, AYA cancer survivors were diagnosed with diverse cancer types and treatment modalities. However, the study had also some limitations that should be taken into account when interpreting the results. Firstly, the findings may not be generalized to the general AYA population. As adolescents diagnosed with cancer between the ages of 15 and 17 were not included, the results cannot be generalized to this group. The study was mainly conducted among participants of the SURVAYA study. In this study, previous analyses indicated potential underrepresentation of specific subgroups, including males, AYA cancer survivors with a lower SES, those with a different cultural background, those in lower disease stages, and certain tumor types [28]. The educational backgrounds in the current study were notably higher than in the general Dutch population. Consequently, caution should be exercised when extending these findings to the wider AYA population. Secondly, our study did not use validated questionnaires to asses informational needs and sources of support [41], making it complicated to compare with international literature. It appears that the responses in this study also reflects support needs beyond information, including emotional and instrumental needs. Lastly, AYA cancer survivors were asked to fill in an open-ended response informational needs and sources from the past. Informational needs can vary over time. Preferably, a longitudinal study should be conducted that assesses informational needs across different time points.

### Implications

Information and support services from the internet are becoming increasingly important for cancer survivors, especially for AYA cancer survivors. Kanker.nl [27] is the most important source for cancer-related information in the Netherlands offering a wide range of information and support sources. We found that AYA cancer survivors have general and AYA-related informational needs as well. The general information about treatments and cancer types can be addressed on the Kanker.nl website, but age-specific AYA information and services were, until recently, not yet available. In collaboration with AYAs with lived experiences, information on diverse key themes that have emerged are bundled in the “young and cancer” platform (https://www.kanker.nl/jong), such as fertility and pregnancy, dealing with the people around you, school and education, and work and reintegration. This “young and cancer” platform also provides separate information for relatives (e.g., parents, partners, siblings, and friends). AYA cancer survivors reported difficulties in finding peers of their own age with similar experiences. On the “young and cancer” platform, AYA with lived experience share their experiences in blogs, interviews, and podcasts, and it offers opportunities to participate in online support groups for AYA cancer survivors and their relatives. A “peer finder” tool has been developed to facilitate the search for peers based on information from their personal profile such as age, type of cancer, stage of the disease, and short- and long-term effects. AYA cancer survivors can also ask questions to HCPs on the platform, such as a social worker with specific expertise in the treatment and psychological support of AYA cancer survivors.

Future studies should address the informational needs and communication preferences of subgroups within the AYA population, including lower educated AYA cancer survivors and AYA cancer survivors with diverse cultural backgrounds [42]. Informational needs seem to vary by level of education and to be related to the ability to understand and use information about their health [43]. Previous studies have reported that 30% of the AYA cancer survivors have poor health literacy [44]. The aforementioned online platform contains, text and various formats such as images, audio, and video. Alternative methods of information provision could be used to meet the specific needs and preferences of diverse cultural background, such as sharing the experiences of these specific groups so that AYA cancer survivors can identify with them and to providing information in different languages.

## Conclusion

AYA cancer survivors have a need for age-specific information, on themes relating to fertility and pregnancy, family and children, work and reintegration, peers with cancer, and intimacy and sexuality. To effectively address their informational needs, support services must tailor their resources and interventions to AYA cancer survivors and their relatives, including age-specific information and services and opportunities to connect with peers. More research is needed of subgroups such as those with lower levels of education and different cultural background, in order to tailor AYA care to a wider AYA population.

## Supplementary Information

Below is the link to the electronic supplementary material.

Supplementary file1 (DOCX 192 KB)

## Data Availability

The data presented in this study are available on reasonable request from the corresponding author. The data are not publicly available due to privacy issues.

## Abbreviations

AYAs: Adolescents and Young Adults
HCPs: Healthcare professionals
GP: General practitioner
DCS: Dutch Cancer Society
