# Supplementary material for: Identifying the informational needs and sources of support of Adolescent and Young Adult (AYA) cancer survivors to inform the development of a digital platform
Source: J Cancer Surviv. 2024 Oct 18;20(2):683–703. doi: 10.1007/s11764-024-01679-z (PMC12989023; doi:10.1007/s11764-024-01679-z)

**Supplementary files**

**Supplemental figure 1.** The Dutch AYA “Young & Cancer” Care Network's AYA anamnesis. The English version of the AYA anamnesis tool is here available: <https://ayazorgnetwerk.nl/anamnese-tool/>


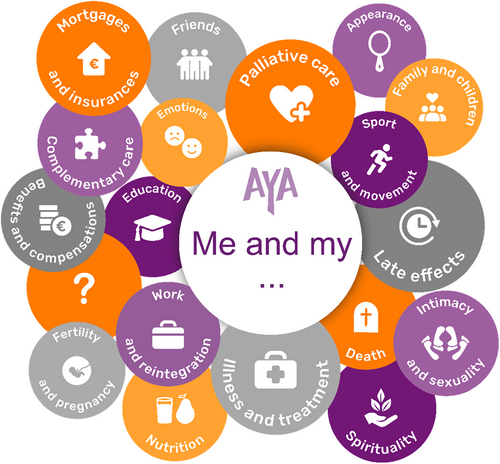

Supplement: Supplementary file 1 — Supplementary file1 (DOCX 192 KB) [file 11764_2024_1679_MOESM1_ESM.docx]
